# Functional Genomics Assistant (FUGA): a toolbox for the analysis of complex biological networks

**DOI:** 10.1186/1756-0500-4-462

**Published:** 2011-10-28

**Authors:** Ignat Drozdov, Christos A Ouzounis, Ajay M Shah, Sophia Tsoka

**Affiliations:** 1 Cardiovascular Division - King's College London (KCL) BHF Centre of Research Excellence - School of Medicine - James Black Centre - 125 Coldharbour Lane, London SE5 9NU - UK; 2 Centre for Bioinformatics - Department of Informatics - School of Natural & Mathematical Sciences, King's College London (KCL) - Strand, London WC2R 2LS - UK; 3 Computational Genomics Unit, Institute of Agrobiotechnology - Centre for Research & Technology Hellas - Thessaloniki - Greece; 4 Donnelly Centre for Cellular & Biomolecular Research - University of Toronto - 160 College Street, Toronto, Ontario M5S 3E1 - Canada

## Abstract

**Background:**

Cellular constituents such as proteins, DNA, and RNA form a complex web of interactions that regulate biochemical homeostasis and determine the dynamic cellular response to external stimuli. It follows that detailed understanding of these patterns is critical for the assessment of fundamental processes in cell biology and pathology. Representation and analysis of cellular constituents through network principles is a promising and popular analytical avenue towards a deeper understanding of molecular mechanisms in a system-wide context.

**Findings:**

We present Functional Genomics Assistant (FUGA) - an extensible and portable MATLAB toolbox for the inference of biological relationships, graph topology analysis, random network simulation, network clustering, and functional enrichment statistics. In contrast to conventional differential expression analysis of individual genes, FUGA offers a framework for the study of system-wide properties of biological networks and highlights putative molecular targets using concepts of systems biology.

**Conclusion:**

FUGA offers a simple and customizable framework for network analysis in a variety of systems biology applications. It is freely available for individual or academic use at http://code.google.com/p/fuga.

## Background

Advances in high throughput data collection and analysis have shown that a discrete biological function can only rarely be attributed to individual molecules [1]. Instead, complex cellular activities can be achieved through a system of interactions between macromolecules such as proteins, DNA, and RNA. Quantitative understanding of these patterns is critical for an in-depth characterization of fundamental principles in cellular biology and pathology.

Network biology is an emerging area of scientific interest aiming at the elucidation of the dynamic structure and pleiotropic function of genes and their products in cellular networks in a systematic and unbiased fashion. The treatment of biological data as graphs, where typically nodes signify cellular entities (e.g. genes, proteins, metabolites) and connections (edges) denote the corresponding functional or physical interactions, is a promising representation in molecular systems biology. Successful applications of network biology have led, for instance, to the classification of breast cancer at the interactome level [2], the characterization of signaling pathways in gastric cancer [3] and the delineation of fundamental organizational principles in metabolic networks [4]. In addition, integration of gene expression and network topological properties have led to more efficient methods for novel biomarker identification in critical conditions such as heart failure [5] and cancer [2].

Analysis of topological features and dynamic properties of cellular networks remains a highly active area of research. Indeed, several tools have been developed to address the need for system-wide analysis (NeAT [6], Systems Biology Toolbox [7]) or visualization (Cytoscape [8], BioLayout [9]). Nonetheless, the adoption of such approaches within the wider biomedical community has been rather limited, possibly due to challenges posed by the integration of experimental (e.g. microarray) and computational (e.g. databases) platforms [10]. Therefore, it is desirable that network-driven pipelines become more accessible to end users, thus facilitating the transformation of information hidden in multi-dimensional datasets into useful hints for the discovery of biomarkers and therapeutic targets.

## Implementation

**Figure 1:**
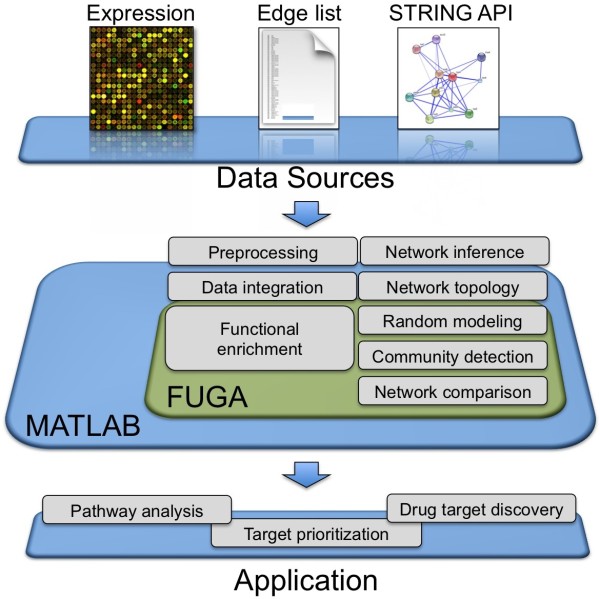
**An overview of the most prominent FUGA features**. FUGA is used for gene regulatory network inference, random modeling, network topology analysis, clustering, and network comparison. It is also possible to import biological networks from the STRING web-based database. Integration of biomedical data such as gene expression with interactome information may facilitate molecular pathway analysis, network-based target prioritization, and drug target discovery.

We have developed a system called Functional Genomics Assistant (FUGA), a MATLAB toolbox for the inference of cellular networks, graph topology analysis, random network simulation, network clustering, and functional enrichment (shown schematically in Figure 1). The toolbox is easily customizable and scalable to networks with thousands of nodes and millions of edges. Additionally, FUGA can integrate high throughput datasets into a unified framework, within which other applications can be embedded. Our objective in designing FUGA has been to simplify network analysis concepts and techniques for end users, by providing intuitive MATLAB functions for complex system exploration and network analysis.

MATLAB was the language of choice for the development of FUGA, due to its matrix- and vector-based architecture, simple syntax, and powerful graphics. The code is designed to run on Unix or Mac machines; advanced users may compile sources for other platforms. Overall, FUGA is seamlessly integrated into MATLAB, so as to permit extensive analytical and visualization operations.

## Results and Discussion

Recently, FUGA functionality was applied on experimentally derived datasets to define a population-based miRNA signature of type 2 diabetes [11], elucidate the expression patterns of iron regulatory protein 2 (IRP2) [12], and characterize genome-wide expression patterns in physiological cardiac hypertrophy [13]. The current FUGA release contains 137 functions and the main operating features are described briefly below.

### Network reconstruction

Biological networks can be inferred with FUGA from computationally or experimentally derived datasets (e.g. BLAST similarity matrices, microarray data) using any form of similarity measure (e.g. Pearson Correlation Coefficient or PCC) to define pairs of nodes. It is also possible to import network edge lists from a text file or download interactomes of interest from the STRING database of known or predicted protein-protein interactions [14] via a web API. In the future releases, FUGA will support a wider range of public interaction databases. Network graphs are typically undirected and may be weighted. Network-specific information, such as node labels and attributes are stored as MATLAB objects for further access.

### Global and local network topology

Complex interactions between constituents that regulate phenotypic diversity necessitate the study of a biological system in the context of the entire interactome, rather than just the over- or under- expression of individual entities. FUGA provides access to global network parameters such as shortest path, diameter, or link density in the interaction network. Topological features such as node betweenness and clustering coefficients can also be computed by accessing the Markov clustering (MCL) toolset [15] through the FUGA interface. To compare against random effects in large networks with hundreds of nodes and thousands of edges from high-throughput experiments, FUGA can construct random networks using link rewiring and the Erdos-Rényi model, or it can build a random modular graph, as reported elsewhere [16]. For comparing two or more networks, FUGA implements simple network similarity estimations using the Jaccard index and handles boolean set operations such as network union, intersection, and difference.

### Network clustering

Because functionally related genes or proteins tend to co-localize in network vicinity, FUGA offers to identify the modular network structure via the MCL [15] or SPICi protocols [17]. Additionally, FUGA implements a greedy algorithm for community detection that uses network modularity as a measure of community structure [18], as well as several functions for spectral graph partitioning [19]. Such unsupervised algorithms are well adapted to large biological networks and may uncover previously undetected interactions.

### Biological interpretation

By default, FUGA works with the ENSEMBL database [20] to annotate network nodes using all major function classification schemes (e.g. Gene Ontology [21] [GO] Biological Process [BP], Molecular Function [MF], Cellular Component [CC], GOSlim, or Reactome pathway terms). In addition, it is possible to define a custom annotation schema such as gene-disease associations. Cluster enrichment is performed by calculating the hypergeometric probability between inter- and intra- cluster gene counts assigned to *a priori*-defined terms. To facilitate biological discovery, nodes can be explored by merit of their topologies and subsequently linked to databases such as ENTREZ, GeneCards, or UniProt. Similar topological analyses have been previously used to uncover high-quality therapeutic targets in psoriasis [22] and identify putative cancer-associated genes [23], for instance. We illustrate biological discovery with FUGA through an example (see below).

### Visualization through Cytoscape

FUGA provides direct access to the Cytoscape graph visualization software. Networks and node attributes, including clusters and topologies, can also be exported to a text file for subsequent exploration with other, user-defined network visualization software.

### Extensibility

MATLAB's interactive environment allows flexible and simple addition of new functionalities to FUGA by expanding the existing framework. For example, additional network statistics or clustering algorithms can be implemented using MATLAB matrix operations and the existing FUGA network object architecture.

### Comparison with other toolboxes

Similar toolboxes have been developed in MATLAB for systems biology related analysis. These include MATLABBGL [24] and the Brain Connectivity Toolbox (BCT) [25], as well as the bioinformatics toolbox developed by Mathworks. MATLABBGL uses the Boost Graph library to efficiently analyze large sparse graph structures. The BCT package is designed to quantify centrality and structure of brain networks. The bioinformatics toolbox from Mathworks has several functions for graph analysis (e.g. connected components, shortest paths), but its functionality is limited, as it may not scale up well for larger genome-wide networks. A number of non-MATLAB tools are available for network visualization and analysis, including NATbox [26] and NeAT [27]. Each tool has a distinct set of features which are highlighted in Table 1. The FUGA toolbox provides a broader range of graph theory functions and integrates expression analysis, functional annotation, and network visualization. The current FUGA release 2.9.4 contains 137 functions. As such, it provides an important contribution to network biology applications and related biomedical data analyses.

**Table 1:** Comparison of network analysis tools.

Tool	FUGA	BIT	BGL	BCT	NATbox	igraph	NeAT	NWB	IN	GG
**Interface**	** MATLAB **	** MATLAB **	** MATLAB **	** MATLAB **	**R**	**C/R/Python**	**Web-based**	**Java**	**Java**	**Java**

User-defined networks	✓	✓	✓	✓	✓	✓	✓	✓	✓	✓

Curated pathway/network content: API	✓						✓		✓	✓

Computational network reconstruction	✓				✓				✓	✓

Statistical network analysis	✓	✓	✓	✓	✓	✓	✓	✓	✓	✓

Biological enrichment/annotation	✓				✓		✓		✓	✓

Expression analysis	✓				✓				✓	✓

BIT = MATLAB Bioinformatics Toolbox, BGL = MATLABBGL, BCT = Brain Connectivity Toolbox, NWB = Network Workbench, IN = Ingenuity Pathways Analysis, GG = GeneGo.

### Example

**Figure 2:**
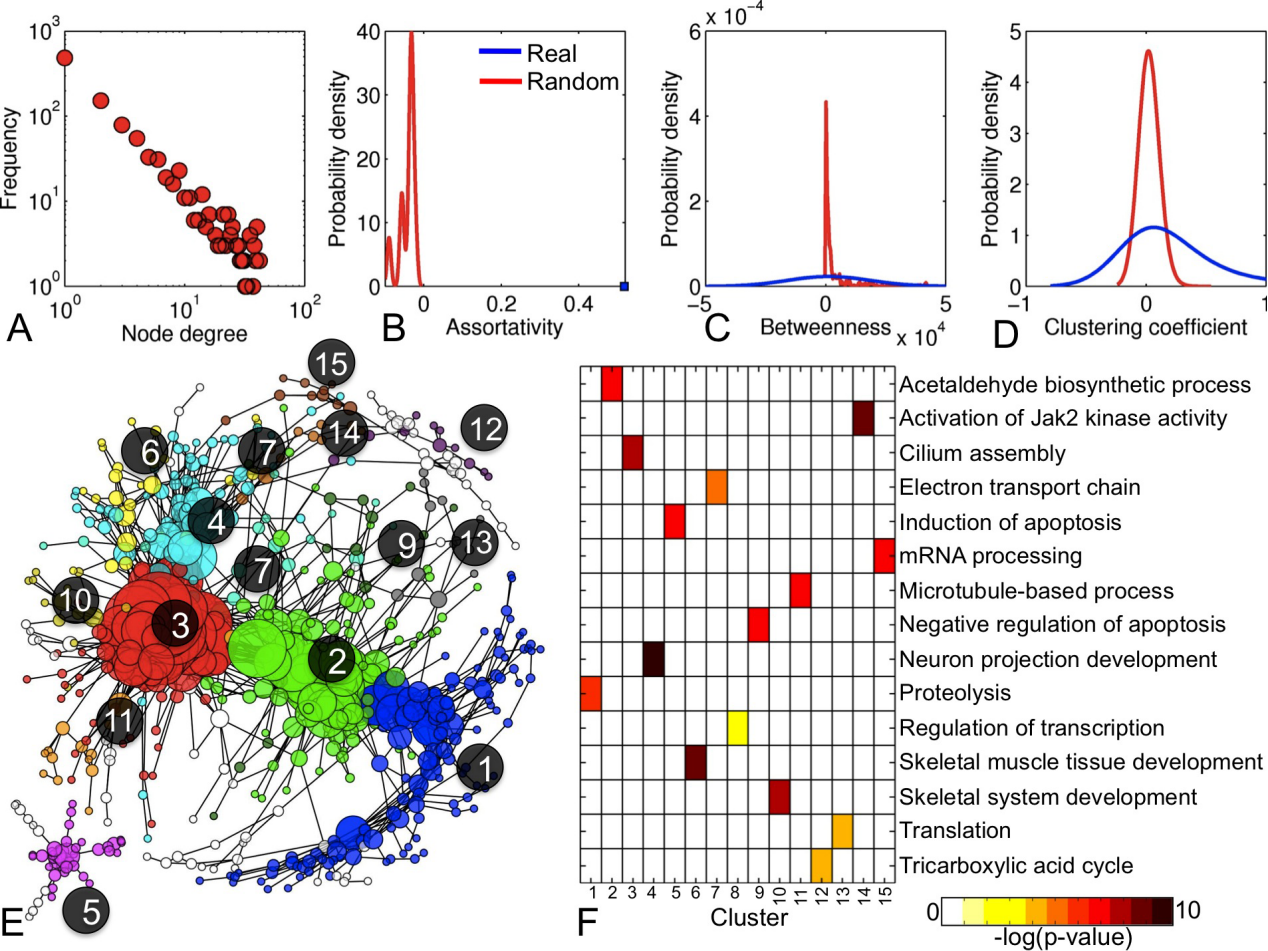
**Example analysis of cardiac hypertrophy using FUGA**. A-D) Comparison of topological network properties in the gene co-expression network and 10 random networks generated using the Maslov-Sneppen rewiring model [16]. **E) **Cytoscape visualization of the gene co-expression network in cardiac hypertrophy. Each node represents a gene and links represent a co-expression. Node colors reflect gene cluster assignment (numerical labels) as determined by the Louvain algorithm and node sizes are proportional to node degrees. The largest 15 gene clusters are visualized. **F) **Enrichment of each network cluster for over-represented Gene Ontology Biological Process terms.

We illustrate the functionality of FUGA by interrogating time-course transcriptional profiles of failing mouse hearts (ArrayExpress: E-MEXP-105 [28]). First, PCCs for all possible gene pairs across all phenotypes were computed and gene pairs with absolute PCC≥0.90 were retained and visualized as an un-weighted, undirected network. The network contained 1018 genes and 2324 links. Average node degree was 4.6, network diameter was 21, and graph architecture was determined to be scale free (Figure 2A). Topological features of the network (assortativity, betweenness, clustering coefficients) were non-random (Figure 2B-D). Then, the greedy method for community detection [18] was used to identify modules of co-expressed genes (Figure 2E). The network and its attributes were visualized with Cytoscape and biological enrichment was performed to identify disease-specific over-represented GO-BP terms (Figure 2F). The above analyses were executed in under 5 minutes in the MATLAB command line prompt on 2.53 GHz Intel Core 2 Duo machine with 4 GB RAM.

## Conclusion

FUGA is an extensible and versatile framework for network analysis in a variety of systems biology applications. While currently FUGA implements the most widely used graph theoretic approaches, future plans include the development of novel centrality and clustering algorithms. We also aim to integrate additional biological annotation repositories to facilitate analysis of networks derived from heterogeneous biological experiments. The FUGA project is an ongoing effort that should facilitate the dissemination of graph theoretic approaches across the wider biomedical community.

## Availability and requirements

Project name: FUGA

Project web page: http://code.google.com/p/fuga/

Operating System: Mac OS X/Linux/Windows

Programming language: MATLAB 7.8.0 or higher/C/C++

Other requirements: None

License: GPL

Any restrictions on use by non-academics: License needed

## Authors' contributions

ID created the software, and drafted the manuscript. CAO edited the manuscript and advised on software design. AMS coordinated the project design and edited the manuscript. ST coordinated the project design and wrote the manuscript. All authors read and approved the manuscript.

## References

[B1] Barabasi AL Oltvai ZN Network biology: understanding the cell's functional organization Nat Rev Genet 2004 5 2 101 113 10.1038/nrg1272 14735121

[B2] Chuang HY Lee E Liu YT Lee D Ideker T Network-based classification of breast cancer metastasis Mol Syst Biol 2007 3 140 17940530 10.1038/msb4100180 PMC2063581

[B3] Aggarwal A Guo DL Hoshida Y Yuen ST Chu KM So S Boussioutas A Chen X Bowtell D Aburatani H Topological and functional discovery in a gene coexpression meta-network of gastric cancer Cancer Res 2006 66 1 232 241 10.1158/0008-5472.CAN-05-2232 16397236

[B4] Guimera R Nunes Amaral LA Functional cartography of complex metabolic networks Nature 2005 433 7028 895 900 10.1038/nature03288 15729348 PMC2175124

[B5] Azuaje F Devaux Y Wagner DR Coordinated modular functionality and prognostic potential of a heart failure biomarker-driven interaction network BMC Syst Biol 2010 4 60 10.1186/1752-0509-4-60 20462429 PMC2890499

[B6] Brohee S Faust K Lima-Mendez G Vanderstocken G van Helden J Network Analysis Tools: from biological networks to clusters and pathways Nat Protoc 2008 3 10 1616 1629 10.1038/nprot.2008.100 18802442

[B7] Schmidt H Jirstrand M Systems Biology Toolbox for MATLAB: a computational platform for research in systems biology Bioinformatics 2006 22 4 514 515 10.1093/bioinformatics/bti799 16317076

[B8] Shannon P Markiel A Ozier O Baliga NS Wang JT Ramage D Amin N Schwikowski B Ideker T Cytoscape: a software environment for integrated models of biomolecular interaction networks Genome Res 2003 13 11 2498 2504 10.1101/gr.1239303 14597658 PMC403769

[B9] Theocharidis A van Dongen S Enright AJ Freeman TC Network visualization and analysis of gene expression data using BioLayout Express(3D) Nat Protoc 2009 4 10 1535 1550 10.1038/nprot.2009.177 19798086

[B10] Gonzalez-Angulo AM Hennessy BT Mills GB Future of personalized medicine in oncology: a systems biology approach J Clin Oncol 2010 28 16 2777 2783 10.1200/JCO.2009.27.0777 20406928 PMC2881854

[B11] Zampetaki A Kiechl S Drozdov I Willeit P Mayr U Prokopi M Mayr A Weger S Oberhollenzer F Bonora E Plasma microRNA profiling reveals loss of endothelial miR-126 and other microRNAs in type 2 diabetes Circ Res 2010 107 6 810 817 10.1161/CIRCRESAHA.110.226357 20651284

[B12] Maffettone C Chen G Drozdov I Ouzounis C Pantopoulos K Tumorigenic properties of iron regulatory protein 2 (IRP2) mediated by its specific 73-amino acids insert PLoS One 2010 5 4 e10163 10.1371/journal.pone.0010163 20405006 PMC2854138

[B13] Drozdov I Tsoka S Ouzounis CA Shah AM Genome-wide expression patterns in physiological cardiac hypertrophy BMC Genomics 2010 11 557 10.1186/1471-2164-11-557 20937113 PMC3091706

[B14] Jensen LJ Kuhn M Stark M Chaffron S Creevey C Muller J Doerks T Julien P Roth A Simonovic M STRING 8--a global view on proteins and their functional interactions in 630 organisms Nucleic Acids Res 2009 37 Database D412 416 10.1093/nar/gkn760 PMC2686466 18940858

[B15] Enright AJ Van Dongen S Ouzounis CA An efficient algorithm for large-scale detection of protein families Nucleic Acids Res 2002 30 7 1575 1584 10.1093/nar/30.7.1575 11917018 PMC101833

[B16] Maslov S Sneppen K Specificity and stability in topology of protein networks Science 2002 296 5569 910 913 10.1126/science.1065103 11988575

[B17] Jiang P Singh M SPICi: a fast clustering algorithm for large biological networks Bioinformatics 2010 26 8 1105 1111 10.1093/bioinformatics/btq078 20185405 PMC2853685

[B18] Blondel VD Guillaume J-L Lambiotte R Lefebvre E Fast unfolding of communities in large networks J Stat Mech 2008 P10008

[B19] Chen D Burleigh GJ Fernandez-Baca D Spectral partitioning of phylogenetic data sets based on compatibility Syst Biol 2007 56 4 623 632 10.1080/10635150701499571 17654366

[B20] Birney E Andrews TD Bevan P Caccamo M Chen Y Clarke L Coates G Cuff J Curwen V Cutts T An overview of Ensembl Genome Res 2004 14 5 925 928 10.1101/gr.1860604 15078858 PMC479121

[B21] Ashburner M Ball CA Blake JA Botstein D Butler H Cherry JM Davis AP Dolinski K Dwight SS Eppig JT Gene ontology: tool for the unification of biology. The Gene Ontology Consortium Nat Genet 2000 25 1 25 29 10.1038/75556 10802651 PMC3037419

[B22] Dezso Z Nikolsky Y Nikolskaya T Miller J Cherba D Webb C Bugrim A Identifying disease-specific genes based on their topological significance in protein networks BMC Syst Biol 2009 3 36 10.1186/1752-0509-3-36 19309513 PMC2678983

[B23] Milenkovic T Memisevic V Ganesan AK Przulj N Systems-level cancer gene identification from protein interaction network topology applied to melanogenesis-related functional genomics data J R Soc Interface 2010 7 44 423 437 10.1098/rsif.2009.0192 19625303 PMC2842789

[B24] MatlabBGL http://www.stanford.edu/~dgleich/programs/matlab_bgl/

[B25] Rubinov M Sporns O Complex network measures of brain connectivity: uses and interpretations Neuroimage 2010 52 3 1059 1069 10.1016/j.neuroimage.2009.10.003 19819337

[B26] Chavan SS Bauer MA Scutari M Nagarajan R NATbox: a network analysis toolbox in R BMC Bioinformatics 2009 10 Suppl 11 S14 10.1186/1471-2105-10-S11-S14 19811679 PMC3152789

[B27] Brohee S Faust K Lima-Mendez G Vanderstocken G van Helden J Network Analysis Tools: from biological networks to clusters and pathways Nature protocols 2008 3 10 1616 1629 10.1038/nprot.2008.100 18802442

[B28] Parkinson H Sarkans U Kolesnikov N Abeygunawardena N Burdett T Dylag M Emam I Farne A Hastings E Holloway E ArrayExpress update--an archive of microarray and high-throughput sequencing-based functional genomics experiments Nucleic Acids Res 2011 39 Database D1002 1004 10.1093/nar/gkq1040 PMC3013660 21071405

